# DEAH-box polypeptide 32 promotes hepatocellular carcinoma progression via activating the β-catenin pathway

**DOI:** 10.1080/07853890.2021.1898674

**Published:** 2021-03-17

**Authors:** Xiaoyun Hu, Guosheng Yuan, Qi Li, Jing Huang, Xiao Cheng, Jinzhang Chen

**Affiliations:** State Key Laboratory of Organ Failure Research, Guangdong Provincial Key Laboratory of Viral Hepatitis Research, Department of Infectious Diseases and Department of Radiation Oncology, Nanfang Hospital, Southern Medical University, Guangzhou, China

**Keywords:** Hepatocellular carcinoma, DHX32, EMT, invasion, proliferation, β-catenin

## Abstract

**Purpose:**

Hepatocellular carcinoma (HCC) is refractory cancer with high morbidity and high mortality. DEAH-box polypeptide 32 (DHX32) was upregulated in several types of malignancies and predicted poor prognosis. Herein, we investigated the role of DHX32 in HCC progression.

**Methods:**

The expression of DHX32, β-catenin, and epithelial-mesenchymal transition (EMT)-related makers were determined by Western blot and quantitative real-time PCR assays. Cell proliferation was tested by EdU cell proliferation assay. The effect of DHX32 and β-catenin on cell migration and invasion were detected by wound-healing and Traswell invasion assays. Tumour xenografts were performed to determine the effect of DHX32 on HCC tumour growth.

**Results:**

High level of DHX32 expression was associated with reduced overall survival in HCC patients. DHX32 expression was upregulated in human HCC cells and ectopic expression of DHX32 induced EMT, promoted the mobility and proliferation of HCC cells, and enhanced tumour growth *in vivo*. Silencing DHX32 reversed EMT, inhibited the malignancy behaviors of HCC cells, and suppressed tumour growth. Mechanistically, silencing DHX32 decreased the expression of β-cateninin in nucleus and β-catenin siRNA abrogated DHX32-mediated HCC progression.

**Conclusion:**

DHX32 was an attractive regulator of HCC progression and indicated DHX32 canserve as a potential biomarker and therapeutic target for HCC patients.

## Introduction

Hepatocellular carcinoma (HCC) is the fifth most common cancer in men and the seventh in woman, which is the third leading cause of cancer deaths all around the world [1]. Surgical resection, trans-arterial chemoembolization, and targeted therapy are the common treatments for early-stage HCC [2–4]. However, most HCC patients developed locally recurrence or metastasis after treatments, thus leading to limited benefits and poor outcomes [5,6]. Therefore, further understanding of molecular mechanisms that contributed to HCC progression was important to identify a novel biomarker and therapeutic target for HCC patients.

Human RNA helicases, consisting of a number of the DEAH box proteins, are closely associated with RNA metabolism processes, such as splicing, degradation, transcription, and translation. They are highly conserved enzymes, and thus play an important role in gene expression [7,8]. DEAH-box polypeptide 32 (DHX32, also known as DDX32) is a new member of the DEAH box helicase family and overexpressed DHX32 was observed in several kinds of solid tumours, including colorectal cancer [9,10] and breast cancer [11,12]. The expression of DHX32 was significantly related to clinically pathological features of colorectal cancer, and can be served as a prognostic biomarker for colorectal cancer patients [10]. It was reported that DHX32 overexpression promoted the proliferation and mobility of colorectal cancer cells and augmented β-catenin signaling to enhance angiogenesis in colorectal cancer [9,13]. Moreover, high level of DHX32 expression also predicted poor prognosis in breast cancer patients [11,12]. However, the role of DHX32 in HCC progression remains largely unknown.

In our study, we aimed to explore the role of DHX32 in HCC progression. We found that high level of DHX32 expression negatively correlated with the overall survival in HCC patients and silencing DHX32 inhibited epithelial mesenchymal transition (EMT) and suppressed the migration, invasion, and proliferation of HCC cells. Mechanistically, DHX32-induced HCC progression was regulated by β-catenin pathway. These findings suggest a role of DHX32/β-catenin axis in HCC aggressiveness.

## Materials and methods

### Cell culture

Human hepatocellular carcinoma cell lines Hep3B (hepatocellular carcinoma cell line containing hepatitis B virus), Huh7 (well differentiated human hepatocellular carcinoma cell line containing hepatitis C virus), SNU-182 (hepatocellular carcinoma cell line containing hepatitis B virus), and SNU-387 (pleomorphic hepatocellular carcinoma cell line), human hepatoblastoma cell line HepG2, and LO2 (human immortalized hepatocyte cell line) were purchased from ATCC. All cells were cultured in DMEM that was supplemented with 10% FBS and kept at 37 °C in a cell incubator with 5% CO_2_.

### Cell stable transfection

For DHX32 overexpression experiments, Huh7 cells (1 × 10^5^ cells/well) were seeded in 6-well plates. When grown to 50% confluence, cells were infected with DHX32 (NM_018180) Human Tagged ORF Clone Lentiviral Particle (Origene; Cat: RC209736L3V) or Lenti-ORF control particles of pLenti-C-Myc-DDK-P2A-Puro (Origene; Cat: PS100092V) using its recommending transfection reagent. For the knockdown of DHX32 experiments, Huh7 cells were infected with DHX32 human shRNA Lentiviral Particle (Santa Cruz Biotechnology; Cat: sc-77143-V) or control shRNA Lentiviral Particle. To establish stable cloning cells expressing or silencing DHX32, the infected cells were selected with 1 μg/ml Puromycin 2HCl (Selleck) for approximately 4 weeks. Then, the expression of DHX32 mRNA and protein was determined by RT-PCR and Western blot assays.

### Cell transition transfection

For DHX32 and β-catenin co-transfection experiments, HCC cells were co-transfected with DHX32 lentiviral particles plus β-catenin siRNA I (Signal Silence; Cat: 6225). Control cells were co-transfected with corresponding vector and control siRNA. The transfection was performed with Lipofectamine™ 3000 Transfection Reagent (Invitrogen; Cat: L3000015) according to its protocol. The expression of DHX32 and β-catenin was detected by Western blot and TR-PCR assays.

### Real-time polymerase chain reaction (RT-PCR) assay

Total RNA in HCC cells was extracted with Total RNA Extraction Kit (Solarbio; Cat: R1200) according to its instruction. The concentration of mRNA was assessed using NanoDrop ND‐1000 spectrophotometer. Then, total RNA was mixed with All-in-One cDNA Synthesis SuperMix (Bimake; Cat: B24403) to generate cDNA. RT-PCR assay was performed with 2 × SYBR Green qPCR Master Mix (Bimake; Cat: B21202) in the LightCycler 480 Real-Time PCR System (Roche) in accordance with the manufactures’ protocol. The relative expression of targeted genes was calculated using 2^–ΔΔCt^ method [13] and was normalized to the expression of *ACTB* in HCC cells.

### Western blot analysis

Total protein in HCC cells was extracted with RIPA Lysis Buffer (Beyotime Technology; Cat: P0013C) containing protease inhibitors and phosphatase inhibitors. Nuclear protein was extracted with Nuclear and Cytoplasmic Protein Extraction Kit (Beyotime Biotechnology; Cat: P0027) according to its instruction. Then, the concentration of protein was assessed using a BCA Protein Kit (Invitrogen; Cat: 23227) and total protein was separated by Western blot assay. Bands were detected with Pierce™ ECL Western Blotting Substrate (Thermo Scientific; Cat: 32209). β-actin serves as a loading control.

### EdU cell proliferation assay

EdU cell proliferation assay was conducted to examine the effect of DHX32 and β-catenin on HCC cell proliferation. Huh7 cells (3 × 10^3^) after different transfections were seeded in 96-well plates and maintained for 48 h. Then, cells were further incubated with 10 μM EdU reagent for 2 h and then performed according to the manufacture’s instruction (RiboBio; Cat: C10310-1). The number of EdU-stained cells were analyzed.

### Wound-healing assay

Wound-healing assay was used to detect the effect of DHX32 and β-catenin on HCC cell migration. Huh7 cells (2 × 10^5^) were seeded in 6-well plates and performed with transfection as indicated. When cells grew to nearly 90% confluency, cell monolayers were scratched with 200 μL pipette tips and followed by cell debris removal by PBS wash. Then, cells were cultured in DMEM medium containing 10% FBS for 24 h. Cells were fixed with 4% paraformaldehyde and stained with 0.1% crystal violet. The wound width of cell monolayers was photographed and the area of wound width was calculated with ImageJ software.

### Cell invasion assay

Transwell invasion assay was performed to detect the effect of DHX32 and β-catenin on HCC cell invasion. Huh7 cells (4 × 10^4^) after the indicated transfections were suspended in serum-free medium and seeded into the Transwell inserts (Corning; Cat: 3422) that was pre-coated with diluted Matrigel (Corning; Cat: 356234). Then wells were filled with culture medium and incubated for 24 h. Next, cells were fixed and stained with 0.1% crystal violet. Invaded cells in the downside of the Transwell filters were observed and the number of invaded cells was calculated.

### Tumour xenografts

Huh7 cells with DHX32 silencing or stable DHX32 overexpression were used to perform tumour xenograft assay. In brief, Huh7 cells (5 × 10^6^ cells/mL) were re-suspended in diluted Matrigel (5 mg/mL) and subcutaneously injected into the flank ofmale BALB/c nude mice (6–8 weeks old, 16–20 g, 6 mice in each group). After tumour engraftment, tumour sizes were measured every four days with callipers. Tumour volume was calculated using the formula: volume = π/6 × a × b^2^ (a = longest diameter, b = shortest diameter perpendicular to a). Tumour weight was analyzed at 31 day after inoculation. Tumour-bearing mice were anaesthetized with 0.5% pentobarbital sodium (approximately 200 μL/mouse, intraperitoneal injection, once) before the collection of tumour tissues.

### Statistical analysis

Data are shown as mean ± SEM. Statistical analysis were performed with GraphPad Prism 7.0 software and statistical differences were analyzed by unpaired two-tail Student’s *t* test or One-way analysis of variance (ANOVA) followed by Tukey’s multiple comparisons test. *p* < .05 was considered statistically significant. The correlation between DHX32 and overall survival in HCC patients was determined using Kaplan-Meier Plotter and the medium DHX32 expression (501) was used as a cut-off.

## Results

### DHX32 is upregulated in HCC cells and predicts poor survival in HCC patients

**Figure 1.**
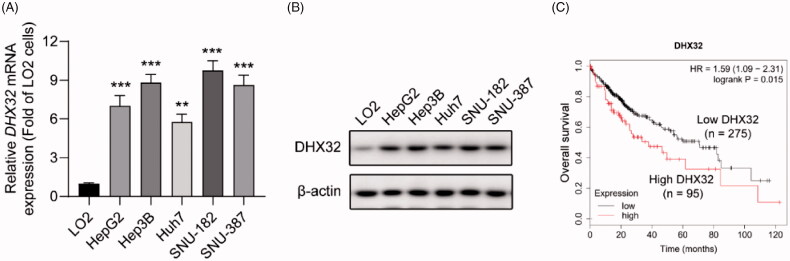
DHX32 expression is upregulated in HCC cells and patients with HCC. (A) The expression of DHX32 mRNA in HCC cells was determined by RT-PCR assay. (B) The expression of DHX32 protein in HCC cells was detected by Western blot assay. (C) Kaplan-Meier plot was used to analyze the overall survival of patients with HCC based on DHX32 expression. The medium DHX32 expression (501) was used as a cut-off. **p < .01 and ***p < .001 compared with LO2 cells.

The expression levels of DHX32 in human HCC cells and human immortalized normal liver LO2 cells were assessed by RT-PCR and Western blot assays. We found that the expression level of DHX32 mRNA and protein were significantly higher in five HCC cell lines, including HepG2 (*p* < .001), Hep3B (*p* < .001), Huh7 (*p* < .01), SNU-181 (*p* < .001), and SNU-387 (*p* < .001) cells, than those in human normal immortalized liver cell LO2 (Figure 1(A and B)). In addition, we also found that high level of DHX32 expression had a negative correlation with overall survival in patients with HCC using Kaplan-Meier Plotter (*p* = .015, Figure 1(C)). Together, these data indicate that DHX32 might serve as a contributor to HCC progression.

### Ectopic expression of DHX32 induces EMT and enhances the migration, invasion, and proliferation of HCC cells

**Figure 2.**
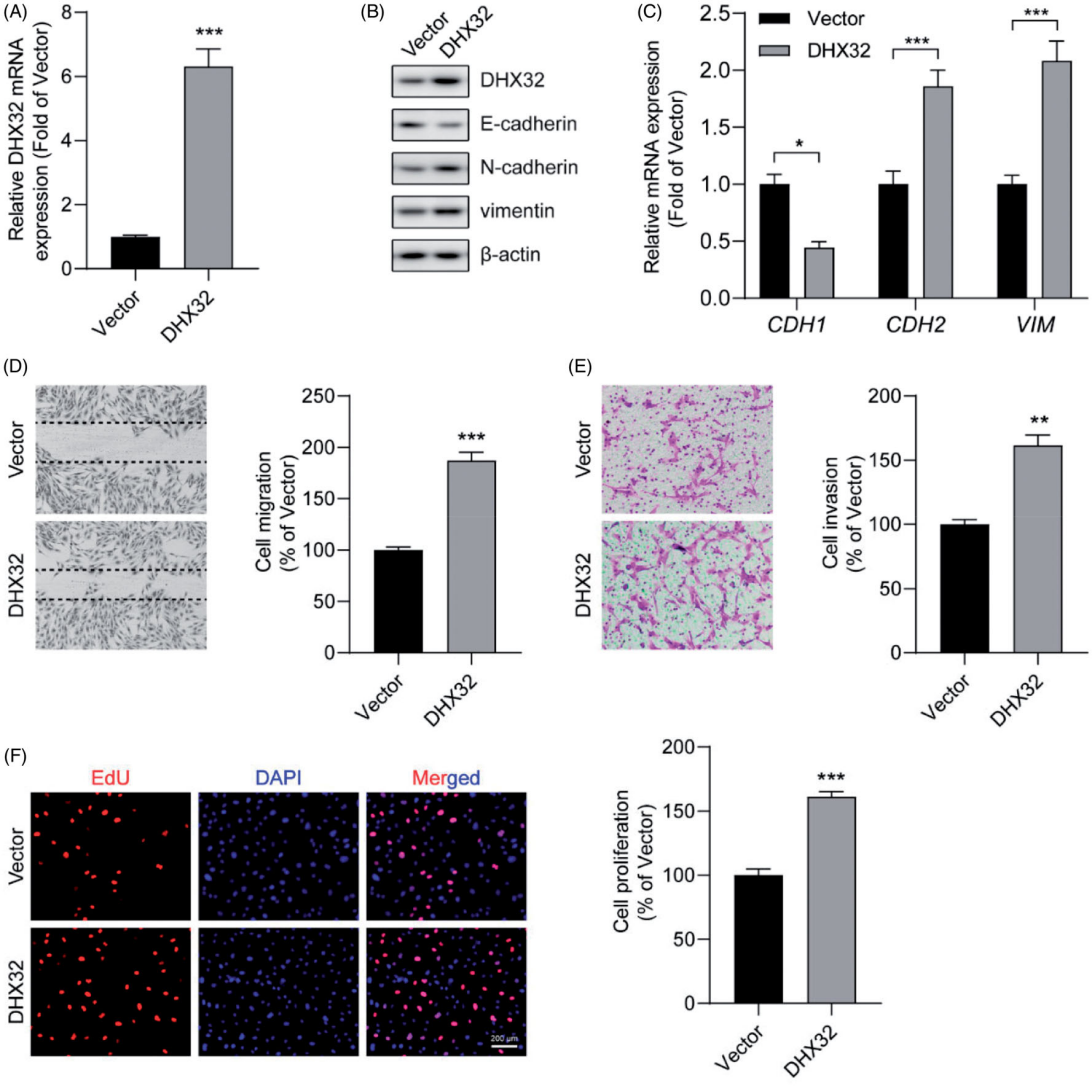
Ectopic expression of DHX32 induces EMT and enhances the migration, invasion, and proliferation of HCC cells. (A) The expression of *DHX32* mRNA in Huh7 cells were determined by RT-PCR assay (B) The expression of DHX32, EMT-related proteins (E-cadherin, N-cadherin, and vimentin) were examined by Western blot assay. (C) The mRNA expression of E-cadherin (*CDH1*), N-cadherin (*CDH2*), and vimentin (*VIM*) in Huh7 cells was determined by RT-PCR assay. (D) Cell migration was tested by wound-healing assay and quantification of cell migration is shown. Magnification, 200 ×. (E) Cell invasion was determined by Transwell invasion assay and quantification the number of invaded Huh7 cells. Magnification, 200 ×. (F)Cell proliferation was detected by EdU cell proliferation assay and quantification of the number of EdU-stained cells. Scale bar, 200 μm. Representative images are shown and data are presented as mean ± SEM (*n* = 5). *p < .05, **p < .01, and ***p < .001 compared with the Vector groups.

To explore whether ectopic expression of DHX32 promoted HCC progression, the migration, invasion, and proliferation of HCC cells were tested. Huh7 cells after the indicated transfections were performed with RT-PCR assay, Western blot assay, wound-healing assay, Trans well invasion assay, and Ed U cell proliferation assay. We confirmed that the expression levels of DHX32 mRNA (*p* < .001) and protein were increased in Huh7 cells transfected with DHX32 lentiviral particles compared with cells transfected with vector lentiviral particles (Figure 2(A and B)). Epithelial-mesenchymal transition is one of the key regulators of invasion and metastasis in HCC [14]. We found that the overexpression of DHX32 induced EMT in HCC cells, leading to increases in the expression of mesenchymal markers N-cadherin and vimentin and reduction in the expression of epithelial marker E-cadherin (Figure 2(B and C)). Then, the effect of DHX32 on HCC cell migration and invasion was examined. Wound-healing assay revealed that DHX32 overexpression increased the migration capacity of Huh7 cells and facilitated the closure of wound width of cell monolayers (*p* < .001, Figure 2(D)). We also observed that the overexpression of DHX32 significantly increased the number of invaded HCC cells (*p* < .001, Figure 2(E)). Moreover, we also detected the effect of DHX32 on HCC cell proliferation. EdU cell proliferation assay showed that ectopic expression of DHX32 promoted HCC cell proliferation, as evidenced by much increase in the number of EdU-positive Huh7 cells (*p* < .001, Figure 2(F)). Taken together, our results suggest that the overexpression of DHX32 induces EMT and promotes the mobility and growth of HCC cells.

### The knockdown of DHX32 reverses EMT and inhibits the migration, invasion, and proliferationof HCC cells

**Figure 3.**
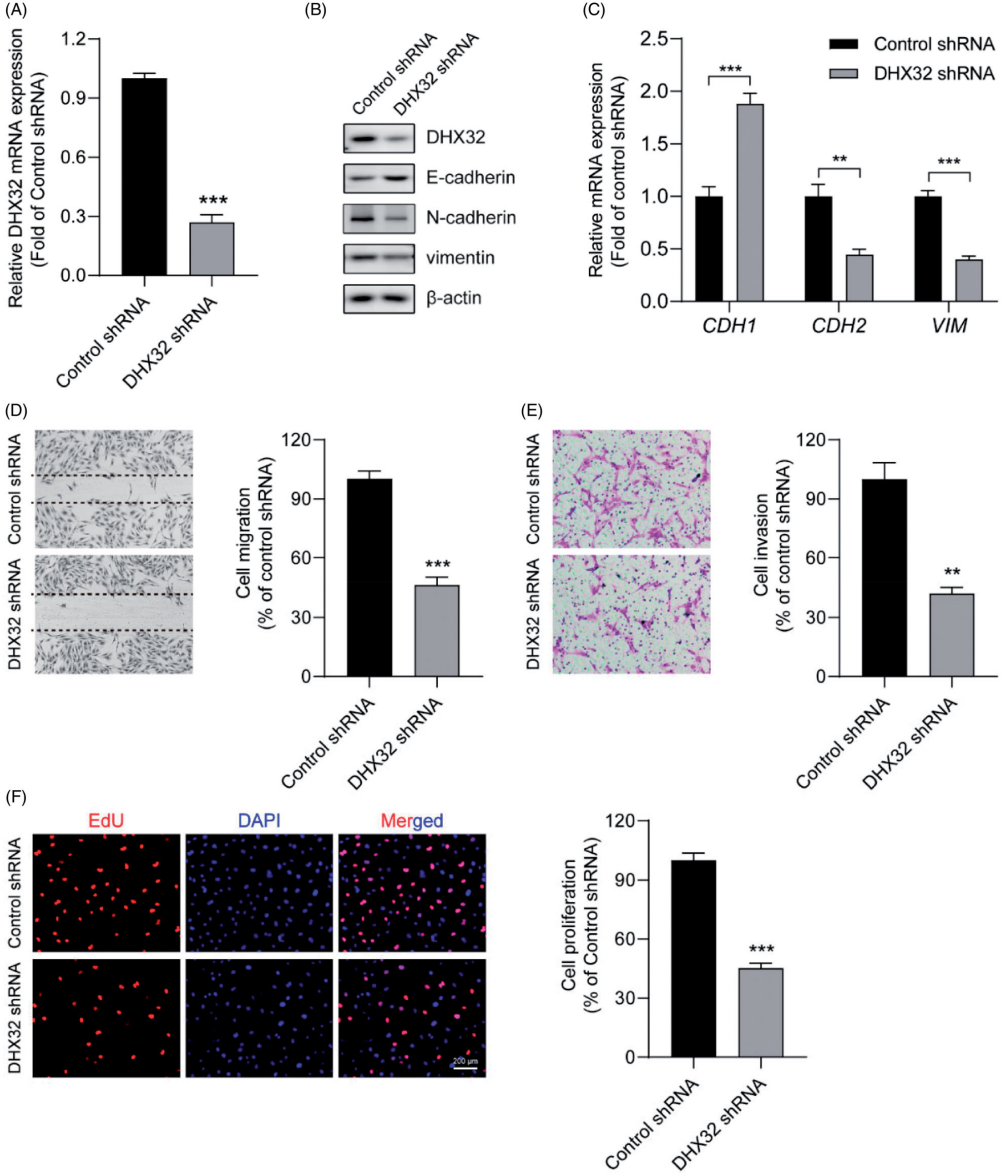
The knockdown of DHX32 reverses EMT and inhibits the migration, invasion, and proliferation of HCC cells. (A) The expression of *DHX32* mRNA in Huh7 cells transfected with control shRNA or DHX32 shRNA were examined by RT-PCR assay. (B) The expression of DHX32, E-cadherin, N-cadherin, and vimentin) were determined by Western blot assay. (C) E-cadherin, N-cadherin, and vimentin mRNA expression in Huh7 cells were measured by RT-PCR assay. (D)The effect of DHX32 shRNA on Huh7 cell migration was examined by wound-healing assay. Magnification, 200 ×. (E) The invasive capacity Huh7 cells transfected with DHX32 shRNA was tested by Trans well invasion assay and the number of invasive cells was calculated. Magnification, 200 ×. (F)The proliferation rate of Huh7 cell proliferation after transfection with DHX32 shRNA was determined by EdU cell proliferation assay. The number of EdU-positive cells was quantified. Scale bar, 200 μm. Representative images are shown and data are presented as mean ± SEM (*n* = 5). **p < .01 and ***p < .001 compared with the Control shRNA groups.

Next, we further investigated the effect of DHX32 knockdown on HCC progression. The expression of DHX32 in Huh7 cells were stably silenced with its specific shRNA lentiviral particles. RT-PCR and Western blot assays showed that the expression of DHX32 was dramatically decreased in Huh7 cells infected with DHX32 shRNA lentiviral particles (Figure 3(A and B)). Then, whether inhibition of DHX32 suppressed EMT in HCC cells was determined. We found that DHX32 shRNA dramatically increased E-cadherin mRNA and protein expression levels, while decreased the expression of N-cadherin and vimentin in Huh7 cells, which indicated that DHX32 shRNA inhibited EMT (Figure 3(B and C)). Wound-healing assay showed that silencing DHX32 inhibited the closure of wound width of Huh7 cell monolayers (*p* < .001, Figure 3(D)). Transwell invasion assay revealed that the knockdown of DHX32 remarkably reduced the invasive capacity of HCC cells (*p* < .01, Figure 3(E)). In addition, we found that DHX32 shRNA significantly decreased the number of EdU-positive Huh7 cells compared with cells transfected with control shRNA (*p* < .001, Figure 3(F)). Thus, these data suggest that silencing DHX32 suppresses EMT and inhibits the migration, invasion, and proliferation of HCC cells.

### DHX32 regulates the activation of ***β***-catenin pathway in HCC cells

**Figure 4.**
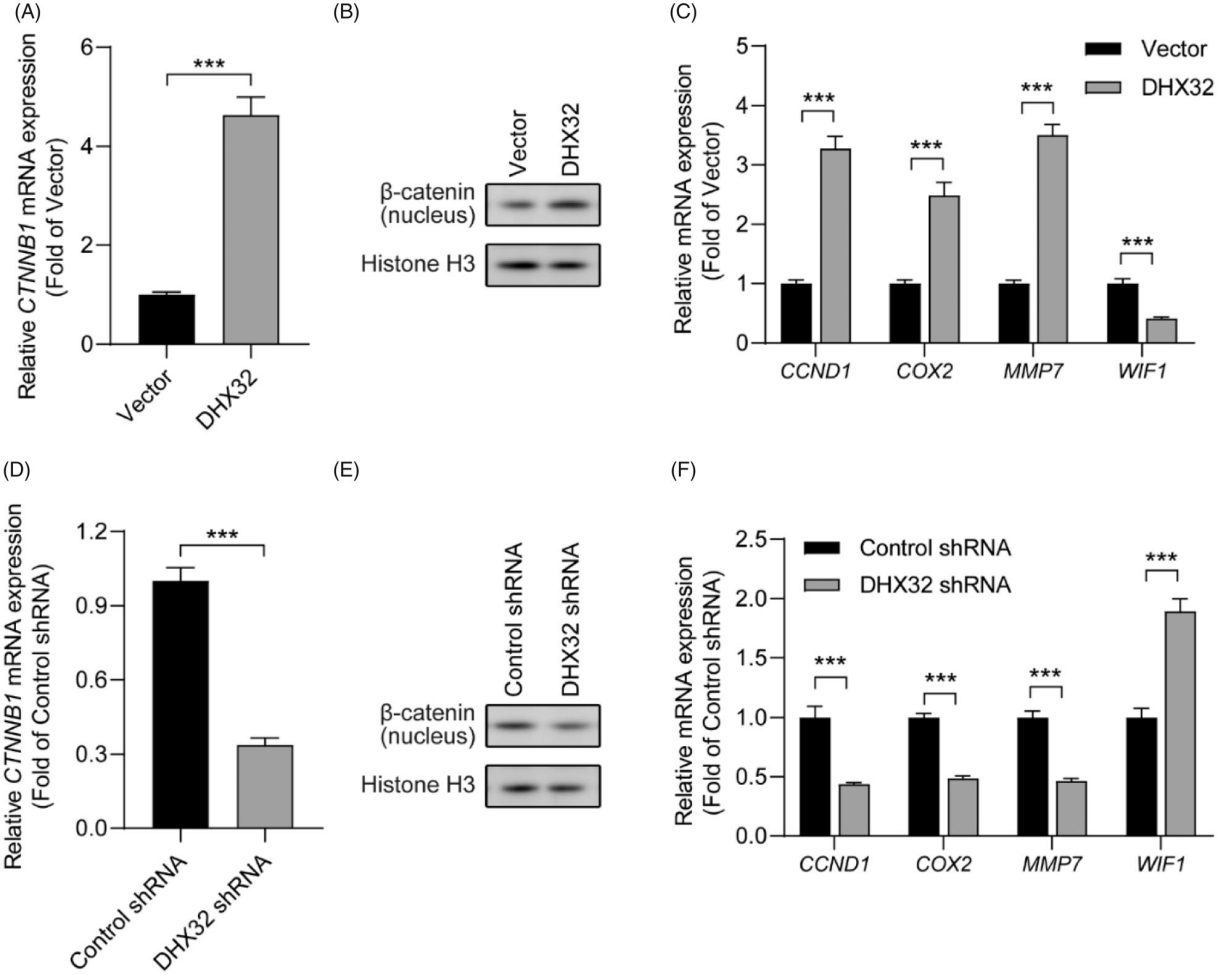
DHX32 regulates the activation of β-catenin pathway in Huh7 cells. (A) The expression of β-catenin in DHX32-overexpressing HCC cells was determined by RT-PCR assay. (B) The protein level of β-catenin in nucleus of Huh7 cells was detected by Western blot assay. (C) The mRNA expression of β-catenin pathway target genes (*CCND1*, *COX2*, and *MMP7*) and *WIF1* in DHX32-overexpressing Huh7 cells was examined by RT-PCR assay. (D)RT-PCR assay for the mRNA expression of Huh7 cells with DHX32 knockdown. (E) The effect of DHX32 shRNA on β-catenin expression in nucleus of Huh7 cells was determined by Western blot assay. (F**)** RT-PCR assay was used to detect the mRNA expression of*CCND1*, *COX2*, *MMP7*, and *WIF1* in Huh7 cells transfected with DHX32 shRNA. Data are presented as mean ± SEM (*n* = 5). *p < .05, **p < .01, and ***p < .001 compared with the Vector- or Control shRNA-transfected groups.

We next investigated the mechanisms of DHX32-medaited EMT and aggressiveness in HCC cells. Since various signalling pathways are involved in EMT and tumour progression, we traced the β-catenin pathway. We found that the overexpression of DHX32 increased *CTNNB1* mRNA expression in Huh7 cells (*p* < .001, Figure 4(A)). Then, we performed Western blot assay to determine whether DHX32 increased the expression β-catenin in nucleus, which is responsible for the activation of β-catenin pathway. We found that the expression of β-catenin in nucleus was much higher in Huh7 cells after transfection with DHX32 lentiviral particle than vector (Figure 4(B)). RT-PCR assay revealed that ectopic expression of DHX32 upregulated the mRNA expression of *CCND1*, *COX2*, and *MMP*7 that were the target genes of the β-catenin pathway (Figure 4(C)). DHX32 overexpression also reduced the expression of WIF1, a negative regulator of Wnt/β-catenin pathway (*p* < .001, Figure 4(C)). Moreover, we further determined whether DHX32 knockdown inactivated the β-catenin pathway in Huh7 cells. We found that silencing DHX32 decreased the expression of *CTNNB1* mRNA and decreased the expression of β-catenin in nucleus of Huh7 cells (*p* < .001, Figure 4(D and E)). In addition, RT-PCR assay further confirmed that the knockdown of DHX32 decreased the mRNA expression of *CCND1*, *COX2*, and *MMP7*, while increased the expression of *WIF1* mRNA in HCC cells (*p* < .001, Figure 4(F)). Together, these findings suggest that DHX32 could activate β-catenin pathway in HCC cells *via* promoting the expression of β-catenin in nucleus.

### β-catenin siRNA abrogates DHX32-induced EMT, migration, invasion, and proliferation in HCC cells

**Figure 5.**
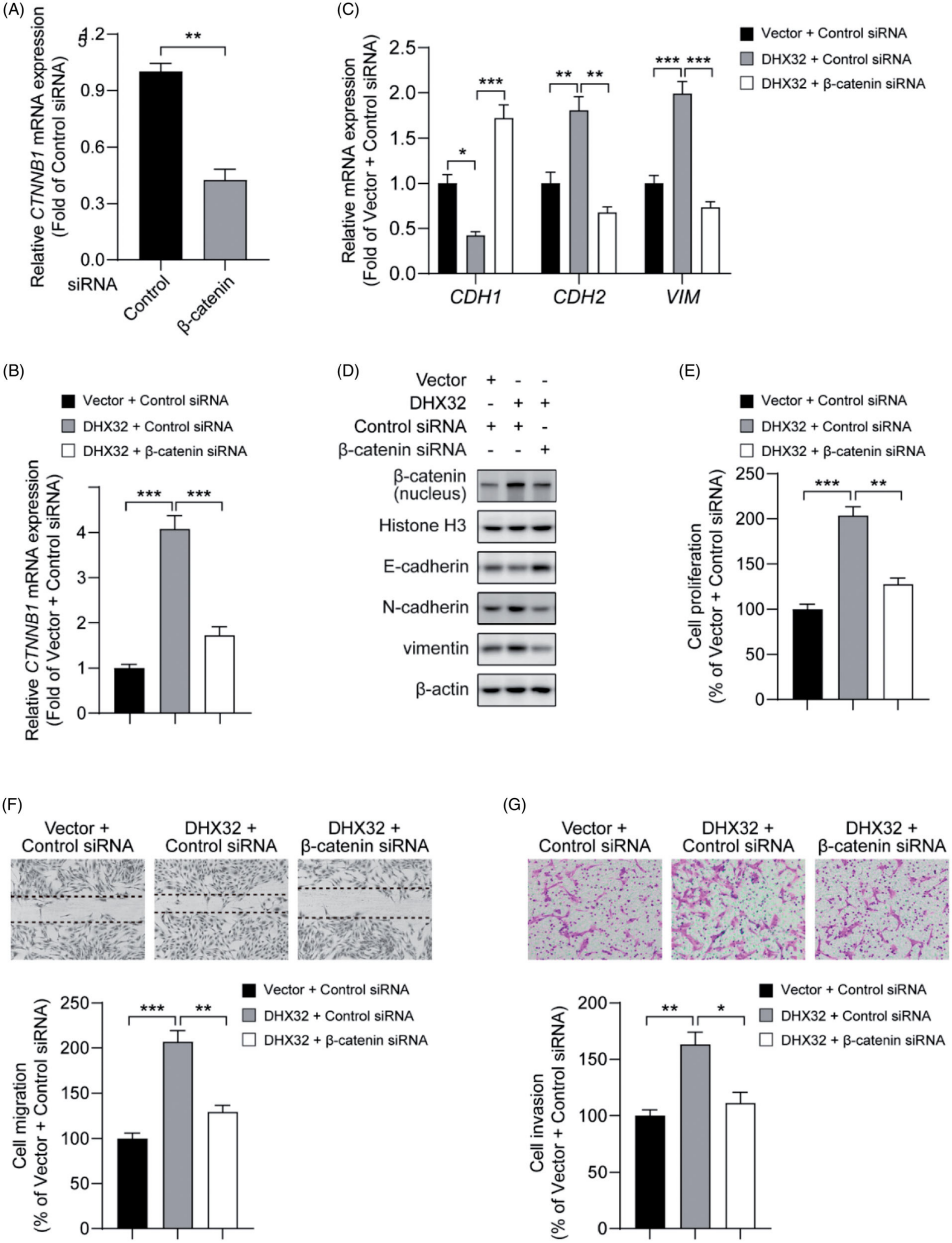
Silencing β-catenin inhibits DHX32-induced HCC progression. (A) RT-PCR assay for the expression of β-catenin (*CTNNB1*) mRNA in Huh7 cells. (B) RT-PCR assay was used to detect the expression of β-catenin (*CTNNB1*) mRNA in Huh7 cells after co-transfection with DHX32 and β-catenin siRNA. (C)Representative blot of the expression of β-catenin in nucleus, E-cadherin, N-cadherin, and vimentin in Huh7 cells with DHX32 overexpression plus β-catenin knockdown. (D) RT-PCR assays were performed to determine the expression of E-cadherin, N-cadherin, and vimentin in Huh7 cells. (E) Quantification of the number of EdU-positive Huh7 cells. (F) Wound-healing assay was used to detect the effect of silencing β-catenin on migration in DHX32-overexpressing HCC cells. Representative images and quantification of cell migration are shown. Magnification, 200 ×. (G) Representative images and quantification of the invaded Huh7 cells after co-transfected with DHX32 lentiviral particle and β-catenin siRNA. Data are presented as mean ± SEM (*n* = 5). *p < .05, **p < .01, and ***p < .001.

Then, we further determined the role of β-catenin in DHX32-mediated mobility and growth in HCC cells. Huh7 cells with stable overexpression of DHX32 were transfected with β-catenin siRNA or control siRNA. We first confirmed that β-catenin siRNA significantly downregulated *CTNNB1* mRNA in Huh7 cells (*p* < .01, Figure 5(A)). Then, RT-PCR assay revealed that DHX32 lentiviral particle significantly increased the mRNA expression of β-catenin in Huh7 cells, which was decreased in cells after co-transfection with DHX32 lentiviral particle plus β-catenin siRNA (*p* < .001, Figure 5(B)). Then, we detected whether DHX32-induced EMT in HCC cells can be reversed by β-catenin siRNA. We found that β-catenin siRNA reversed DHX32-induced EMT in HCC cells, and led to a increase in E-cadherin expression and the decreases in N-cadherin and vimentin expression (Figure 5(C and D)). Expectedly, we found that β-catenin siRNA decreased the ability of DHX32 to increase the proliferation of Huh7 cells and resulted in a significant decrease in the number of EdU-positive cells (Figure 5(E)). Wound-healing assay revealed that the closure of wound width was attenuated in cells after co-transfection with DHX32 lentiviral particle plus β-catenin siRNA, when compared to cells after transfection with DHX32 lentiviral particle (Figure 5(F)). In addition, we also found that β-catenin siRNA reduced the activity of DHX32 to promote the invasion capacity of HCC cells and decreased the number of invasive cells (Figure 5(G)). Taken together, these results demonstrate that DHX32-mediated HCC progression is regulated by β-catenin pathway.

### Inhibition of DHX32 suppresses HCC tumour growth

**Figure 6.**
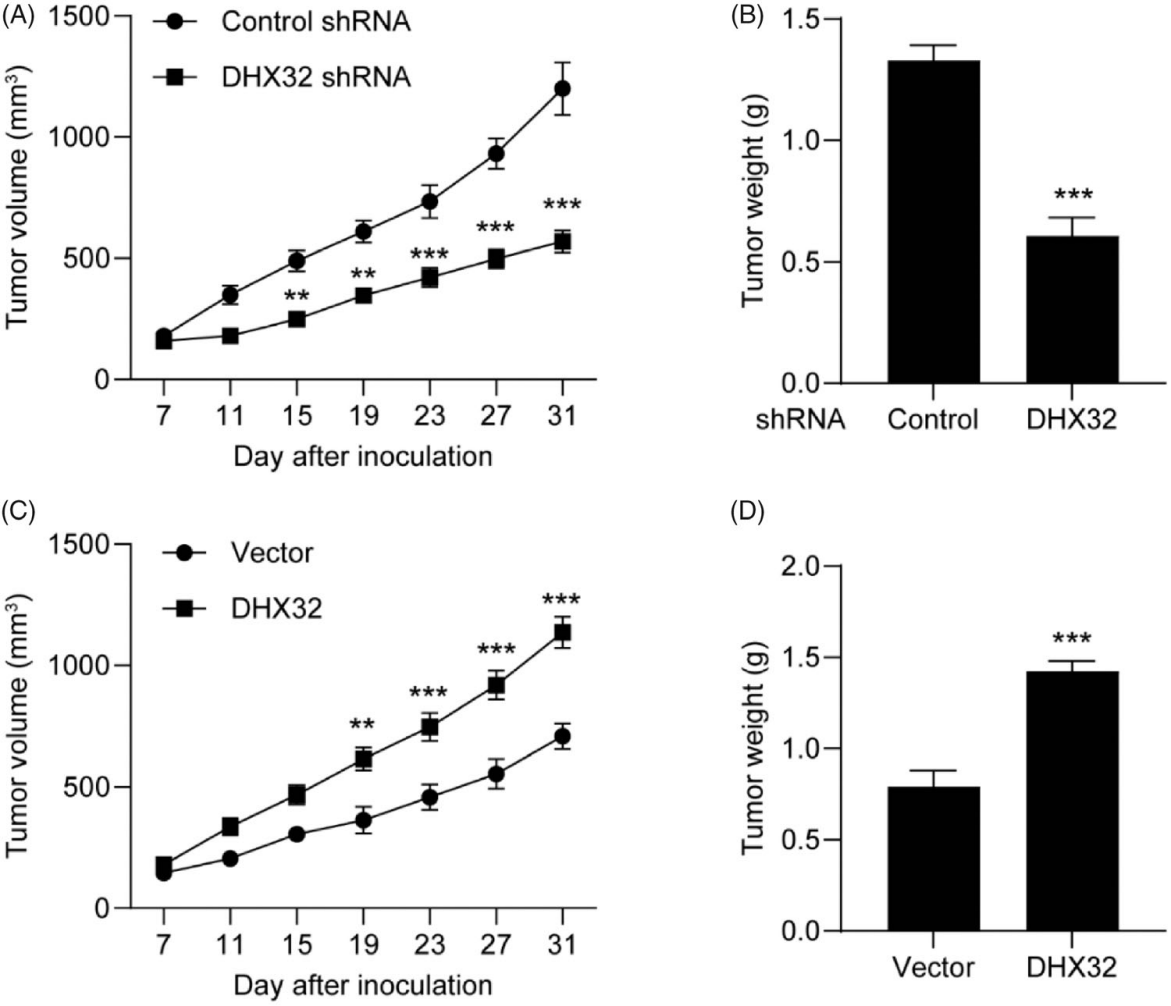
DHX32 inhibition blocks the growth of HCC xenograft tumours. Huh7 cells were stably transfected with DHX32 lentiviral particle or DHX32 shRNA, and inoculated into the flank of male BALB/c Nude mice. Tumour volume and tumour weight were analyzed. (A-B) The volume change (A) and weight (B) of tumours in mice injected with Huh7 cells stably transfected with DHX32 shRNA or control shRNA. (C–D) The volume change (C) and weight (D) of tumours in mice injected with Huh7 cells stably overexpressed DHX32 or vector. Data are presented as mean ± SEM (*n* = 6). **p < .01 and ***p < .001 compared with tumours in mice injected with Huh7 cells stably transfected with control shRNA or vector.

Next, we tested the effect of DHX32 on HCC tumour growth. Huh7 cells after the indicated transfections were subcutaneously injected into the flank of male Balb/c Nude mice and tumour sizes were measured every five days for 4 weeks. Compared to Huh7 cells transfected with control shRNA, silencing DHX32 impaired tumour growth (Figure 6(A)) and decreased the weight of Huh7 xenografted tumours (*p* < .001, Figure 6(B)). Furthermore, we found that ectopic expression of DHX32 promoted HCC tumour growth in comparison with vector-transfected groups (Figure 6(C and D)). Therefore, our *in vivo* study indicates that suppression of DHX32 blocks HCC tumour growth.

## Discussion

The correlation between DHX32 and cancer progression has been explored in several kinds of cancers. DHX32 was reported to function as either a cancer-promoting or a tumour-suppressive gene depending on tumour types, which indicated that the regulation of DHX32 in tumour development might be complex. The expression of DHX32 was upregulated in colorectal cancer (CRC) tissues and remarkably related to local or lymphatic metastasis, differentiation grade, and Dukes’ stage in CRC patients [10]. DHX32 also overexpressed on human CRC cell lines, such as SW480, SW620, and LS174T cells, which enhanced their proliferation and mobility capacities and decreased the chemosensitivity to 5-Fluorouacil [9]. Moreover, DHX32 expression was demonstrated to be negatively correlated with overall survival and disease-free survival in breast cancer patients and could serve as a potential therapeutic target [11]. However, DHX32 was downregulated in acute lymphoblastic leukaemia and might regulate lymphopoiesis [15]. In our study, we found that downregulated expression of DHX32 had a positive correlation with overall survival in patients with HCC. The expression of DHX32 was increased in HCC cell lines than LO2 cells. We also found that the proliferation, migration and invasion of HCC cells were enhanced by the ectopic expression of DHX32, which was reduced by DHX32 silencing. Our study indicated an important role of DHX32 in HCC progression.

Aberrant activation of the canonical Wnt/β-catenin pathway was observed in multiple types of cancer, including HCC [16–18]. Currently, accumulating studies have reported that β-catenin pathway is closely implicated in the growth, EMT, and metastasis of many kinds of solid tumours, such as glioma [19], prostate cancer [20], colorectal cancer [21], and HCC [22]. Inhibiting β-catenin is proven to be a promising therapeutic target for cancers [23–25]. In addition to Wntlig and-dependent activation, the activation of β-catenin pathway can be triggerred in a ligand-independent manner. For example, prospero-related homeobox 1 (PROX1) can enhance the proliferation and decrease the sensitivity to sorafenib in HCC cells through activating the β-catenin pathway [26]. Moreover, Src-homology 2 domain-containing phosphatase 2 (SHP2)promoted the dedifferentiation and enhanced the self-renewal of liver cancer stem cells by augmenting the β-catenin pathway [27]. In our study, we found that the knockdown of DHX32 inhibited the activation of β-catenin and downregulated the target genes of β-catenin pathway. These results indicated that DHX32 might be an upstream regulator of the β-catenin pathway. In addition, β-catenin siRNA can impair DHX32-induced HCC progression. These findings further confirmed that DHX32 promoted the proliferation, migration and invasion *via* amplifying β-catenin pathway. Moreover, Wnt-inhibitory factor-1 (WIF-1), a secreted protein that binds to Wnt proteins and inhibits their activities [28], was downregulated in HCC cells after the transfection of DHX32 overexpressing plasmids. Thus, we could speculate that DHX32 promoted the activation of β-catenin pathway may attribute to DHX32-mediated WIF1 downregulation and the issues needed to be further investigated.

Many biological processes are involved in β-catenin signalling activation, such as decreasing β-catenin degradation and promoting the expression and nuclear translocation of β-catenin [29–31]. PROX1 promoted HCC progression *via* enhancing the expression and nuclear translocation of β-catenin [26]. SHP2 facilitated the nuclear translocation of β-catenin by dephosphorylation of CDC73 and phosphorylation of GSK-3β [27]. In our study, we found that DHX32 siRNA downregulated the mRNA expression of β-catenin and decreased the expression of β-catenin in nucleus of HCC cells. DHX32 either promoted the expression of total β-catenin and enhanced the nuclear translocation of β-catenin, or only promoted the nuclear translocation of β-catenin, or suppressed the expression of WIF1 to activate the β-catenin pathway. However, the underlying molecular mechanisms of DHX32 in augmenting β-catenin signalling remained to be further investigated.

In addition, intrahepatic metastasis and distant metastasis are the leading causes of poor clinical outcomes in HCC patients [6]. In our *in vivo* study, we observed that inhibition of DHX32 suppressed HCC tumour growth. *In vitro* study revealed that the knockdown of DHX32 reversed EMT and blocked the migration and invasion of HCC cells. Thus, more research is needed to evaluate the effect of DHX32 on HCC metastasis in orthotopic HCC models. Moreover, to further confirm the effect of DHX32/β-catenin pathway on HCC proliferation and EMT *in vivo*, we should also detect the expression of proliferation makers, such as PCNA, Cyclin D1, β-catenin expression, and EMT markers including E-cadherin and vimentin in the tumour tissues. In our present study, we drew the major conclusions though using a single cell line and it is better to confirm the conclusion *via* using one more cell line.

## Conclusions

In conclusion, the experimental study reported that overexpressed DHX32 was significantly associated with EMT and increased migration, invasion, and proliferation capacities in HCC cells. Further mechanistic investigations revealed that β-catenin pathway was responsible for DHX32-mediated HCC progression. Our findings demonstrated that targeting DHX32 might be a promising therapeutic strategy for HCC patients.

## Consent for publication

All authors have read the manuscript and given consent for publication.

## Data Availability

All data and materials relevant to this study are available in the article.

## Abbreviations

HCC: Hepatocellular carcinoma
DHX32: DEAH-box polypeptide 32
EMT: Epithelial mesenchymal transition
RT-PCR: Real-time polymerase chain reaction
CRC: Colorectal cancer
PROX1: Prospero-related homeobox 1;
SHP2.: Src-homology 2 domain-containing phosphatase 2
